# A physical map of a BAC clone contig covering the entire autosome insertion between ovine MHC Class IIa and IIb

**DOI:** 10.1186/1471-2164-13-398

**Published:** 2012-08-16

**Authors:** Gang Li, Ka Liu, Shasha Jiao, Haibo Liu, Hugh T Blair, Peng Zhang, Xiaoran Cui, Pingping Tan, Jianfeng Gao, Runlin Z Ma

**Affiliations:** 1 School of Life Sciences, Shihezi University, Xinjiang 832003, China; 2 State Key Laboratory of Molecular Developmental Biology, Institute of Genetics and Developmental Biology, Chinese Academy of Science, Beijing, 100101, China; 3 Institute of Veterinary Animal and Biomedical Sciences, Massey University, Palmerston North, New Zealand; 4 Joint Research Center for Sheep Breeding and Developmental Biology, IGDB-Massey University, Massey, New Zealand; 5 Graduate University of Chinese Academy of Sciences, Beijing, 100149, China; 6 Institute of Genetics and Developmental Biology, Chinese Academy of Science, Beijing, 100101, China

**Keywords:** Ovine, MHC, OLA, Physical map, BAC, Comparative mapping

## Abstract

**Background:**

The ovine Major Histocompatibility Complex (MHC) harbors genes involved in overall resistance/susceptibility of the host to infectious diseases. Compared to human and mouse, the ovine MHC is interrupted by a large piece of autosome insertion via a hypothetical chromosome inversion that constitutes ~25% of ovine chromosome 20. The evolutionary consequence of such an inversion and an insertion (inversion/insertion) in relation to MHC function remains unknown. We previously constructed a BAC clone physical map for the ovine MHC exclusive of the insertion region. Here we report the construction of a high-density physical map covering the autosome insertion in order to address the question of what the inversion/insertion had to do with ruminants during the MHC evolution.

**Results:**

A total of 119 pairs of comparative bovine oligo primers were utilized to screen an ovine BAC library for positive clones and the orders and overlapping relationships of the identified clones were determined by DNA fingerprinting, BAC-end sequencing, and sequence-specific PCR. A total of 368 positive BAC clones were identified and 108 of the effective clones were ordered into an overlapping BAC contig to cover the consensus region between ovine MHC class IIa and IIb. Therefore, a continuous physical map covering the entire ovine autosome inversion/insertion region was successfully constructed. The map confirmed the bovine sequence assembly for the same homologous region. The DNA sequences of 185 BAC-ends have been deposited into NCBI database with the access numbers HR309252 through HR309068, corresponding to dbGSS ID 30164010 through 30163826.

**Conclusions:**

We have constructed a high-density BAC clone physical map for the ovine autosome inversion/insertion between the MHC class IIa and IIb. The entire ovine MHC region is now fully covered by a continuous BAC clone contig. The physical map we generated will facilitate MHC functional studies in the ovine, as well as the comparative MHC evolution in ruminants.

## Background

The mammalian Major Histocompatibility Complex (MHC) harbors genes involved in overall resistance/susceptibility of animals to infectious pathogens, including viral, bacterial, internal and external parasites. Pathogens serve as sources of selection pressure to their host animals, and the hosts are forced to develop effective strategies to fight against the pathogens in various environments. Such co-evolutionary struggles may have left distinct marks in the genome of each species involved, and mammalian MHC regions have been shaped into clusters of immunological gene families by such host-pathogen interactions, probably via functional gene duplications [1-3]. The implications of ovine MHC molecules in providing protection against pathogens [4-8] and the associated structures of the artiodactyl’s MHC region in general have led to a number of studies into the sheep MHC [9-15].

The ovine MHC, also called ovine leukocyte antigen (OLA), is located on the long arm of ovine chromosome 20 (OAR 20q15–20q23) with a similar structure and organization to that of human and other mammals [16]. The literature shows that MHC genes play vital roles in resistance of animals to foot rot [17], parasites [9], and bovine leukemia virus [7]. To date, the majority of studies on the structure and organization of the ovine MHC have focused on the gene content and polymorphism of the class II region [18-23]. Although most loci in the sheep MHC are found to be homologous to their counterparts in the human MHC [12,21,24,25], there are significant differences. Examples of such differences include the *DP* loci in human being replaced by *DY* in sheep [19,21,26,27], and the number of *DQA* loci varying significantly among sheep breeds [20,22,28].

Compared to human and mouse, the structure of the sheep MHC is interrupted by a piece of ~14 Mb autosome insertion, possibly via a hypothetical chromosome inversion (inversion/insertion) in the class II region, similar to that of cattle [24,29-32]. The inversion/insertion constitutes ~25% of ovine chromosome 20, which spliced the MHC class II region into IIa and IIb. The significance of such an insertion in relation to the ovine MHC functions remains unknown. The evolutionary consequence of such an event is also worthy of attention, because some of the ovine-specific MHC loci like *DY,* and *Dsb* are located near the boundary region of the inversion/insertion. We previously constructed a physical map of BAC clone contigs covering the ovine MHC except the autosome insertion region [12,13], and a high accuracy sequence map of sheep OLA was accordingly constructed [14].

With the initial release of sheep whole genome reference sequences by the International Sheep Genomic Consortium (ISGC), much more genome sequence information is now accessible for functional and comparative studies [33]. Nevertheless, the sequence map would serve the research community even better if it is cross-referenced/checked for accuracy in DNA sequence and assembly, at least for some chromosome regions, by an alternative approach. In this regard, the detailed information is still not fully available for the gene structure, organization, and DNA sequence for the ovine chromosome region between OLA class IIa and IIb [12,14,27].

In this paper, we describe the construction of a BAC physical map covering the entire autosome insertion between ovine MHC class IIa and IIb. Because ovine and bovine species share the consensus structure and organization in the entire MHC region [24,29-32], we used comparative approaches to screen a sheep BAC library with 119 bovine oligo nucleotide primers designed from the bovine genomic sequences for the consensus region. The order and overlapping relationship of the identified BAC clones were determined by DNA fingerprinting, BAC-end sequencing, and sequence-specific PCR. A total of 108 effective overlapping BAC clones were selected to fully cover the region between class IIa and IIb. The physical map we constructed will help to generate ovine MHC sequencing map with a high level of accuracy, which in turn will facilitate MHC functional and comparative MHC evolution studies in ruminants.

## Methods

### Comparative design of oligo primers

A BAC library was previously constructed using the genome DNA from a male Chinese merino sheep, with a total of 190,500 BAC clones and an average insert length of 133 kb [12,13]. To screen the BAC library for positive clones in the target genome region between ovine MHC class IIa and IIb, we adapted a comparative strategy to design bovine oligo nucleotide primers using the bovine reference DNA sequences in the consensus genome region [34]. At the time this study was conducted, no sheep genomic sequence was publicly available for the genome region of our concern. Bovine DNA sequences of homologous genes, exon, intron, or partial STS sequences were acquired from the NCBI website (http://www.ncbi.nlm.nih.gov/genome/sts/). Primers were designed along the bovine MHC region between class IIa and IIb, approximately 80–160 kb apart between two neighbor loci using the software Prime Primer 5.0 (Biosoft International, CA). A total of 119 bovine primer pairs were designed for screening the sheep genomic BAC library (Table1).

**Table 1:** Comparative bovine primers used for identification of the positive ovine BAC clones in the genome region between MHC Class IIa and IIb^*^

**Name**	**Gene symbol**	**Primer sequence (5’→3’)**	**Product(bp)**	**Bovine template sequence**	**Positive Ovine** **BAC clones**
S001	*VPS52*	F: ATCAATCAGACGATTCCCAACG	246	UniSTS:279053	12 H14;12I12;12 J14; 12 K14;120P21
		R: ATCAGAAACACAAGCTGCTCCT			
S002	*ZBTB22*	F: TCCTACGACTTACTCCCTCC	250	UniSTS:66823	12I12;12 J14;258 F9; 289 G18
		R: GGGTCAGGTGGTTGTAGTCT			
S003	*KIFC1*	F: GAGACTGTCCGAGACCTGCT	1242	UniSTS:BV104878	170 G9;217 M14;289 G18
		R: CTGTGACTACGCGACGAGC			
S004	*Loc100139397*	F: GGTCATCATGGAGGCAGTCT	756	Exon 6: NC_007324	19 H17
		R: CGTTCTCCTAAGCCATATGC			
S005	*BAK1*	F: CATTGCATGGTGCTAACCGA	293	Exon 6: NC_007324	None
		R: CAAGCTCAGCCTTCCAGAAC			
S006	*IHPK3*	F: ATGTATGAGAGCTTGGCACG	1000	UniSTS:267905	212D3
		R: TCAGCTTGTACTCTTCCAGGG			
S007	*LEMD2*	F: ACGTCTACCGCAACAAGCTG	227	ENSBTAE00000168818: Exon 1	None
		R: GTCTCCGATGTCACCGTAGG			
S008	*Loc790333*	F: GACTGCGAGGTGCCGAAGAA	776	Exon	94 M24;114B22
		R: GTGGACGGCTACACCTGCAA			
S009	*HMGA1*	F: CTCATGCTCTCATTCGGACA	625	ENSBTAE00000364012: Exon 6	57 M5
		R: CAGAACAGGAGGCAATGAGG			
S010	*NUDT3*	F: TGAAGTGGAGAGCCTCACAA	688	ENSBTAE00000213256: Exon 5	14E10;300 G8
		R: CTTCTCAGCAGACGATGGAC			
S011	*COX5B*	F: GTCTCCGTGGTGCGCTCTAT	324	ENSBTAE00000098033: Exon 1,2	130 G21;130 M2;170 K16
		R: GGTGTGGCACCAGCTTGTAA			
S012	*PACSIN1*	F: AAGCCAGCAACAGTAGCAGC	683	ENSBTAE00000336066: Exon 10	253I24
		R: TCGTTACCTGGAGACCAAGC			
S013	*C6orf106*	F: AGTGAGCGGCTGAGAGAGTT	266	ENSBTAT00000048861: Exon 1	None
		R: AACTCGGAGATGAGCACGTC			
S014	*SNRPC*	F: CCAATGATGAGACCTCCTGC	147	ENSBTAT00000034155:Exon 6	119P19;157 K19;223 N7; 227 J17;232 G24
		R: CAGAGTCACAGCACCATGAT			
S015	*TAF11*	F: TGGATGTGTGTGAGAAGTGG	561	ENSBTAT00000022463: Exon 5	194 L19;215 J4;232 G24;234C5
		R: TCATGGTGGAGTATCACAGG			
S016	*ANKS1A*	F: CGAGGAATGGCCACAAAG	894	UniSTS:BV105378	124P23;320A1
		R: ATCGGTCTTGCCAAACAAAG			
S017	*TCP11*	F: ATCAGCGGATCCACTTGTTC	373	ENSBTAT00000022467: Exon 11	24D11
		R: CTGGAGCTCACACACGAGGT			
S018	*DEF6*	F: ACCACCAGCAGCTCCTTCAC	496	ENSBTAT00000036152: Exon 11	21 M13;66I6;124 K16; 193E6;206 L10
		R: CCTGGCTTGCTTGTTGACTC			
S019	*PPARD*	F: GTTCCATGGTCACCTTCTCC	353	ENSBTAT00000023319: Exon 8	28D20;152A4
		R: CCGTGAATCTCGCTTCTCTT			
S020	*TEAD3*	F: CCCATCACAGCTGGATTTTA	145	UniSTS:180986	None
		R: AAATGAAGTACTGTGCCCCC			
S021	*Loc540812*	F: TGCACTGCAACTTCCTGAAC	263	Exon	95D10;119O20;158O6
		R: GCACTGCAGGCTGACTATGA			
S022	*SRPK1*	F: CAGACACTTACAGGACGTGG	273	ENSBTAT00000022396: Exon 11	269D12;285I5
		R: TGAAGACTGGCACATCATGG			
S023	*SLC26A8*	F: ACATCAGCACCGTCAGTCACC	222	UniSTS:476830	26A21;121O15
		R: AGGCGATAGAGGACAAACCACAC			
S024	*MAPK14*	F: GAATGGATAACAAAACACTT	196	UniSTS:279403	26A21;121O15
		R: AGGCGATAGAGGACAAACCACAC			
S025	*MAPK13*	F: AGAAGCTCAATGACAAGGCG	606	UniSTS:269171	121O15;154 M16
		R: TTCCATTCGTCCACTGTGAG			
S026	*BRPF3*	F: GACGCCTGCATCGTATTAGC	575	ENSBTAT00000017711: Exon 1	154 M16;250 L24; 278B11;281D9;300 J5
		R: AGCCAGGTTGCAGATGTCAC			
S027	*PNPLA1*	F: TCCTGAACGCTGTCAACCGA	449	ENSBTAT00000055658: Exon 7	78 M7;153 F9;268E18; 319O4;337 K13
		R: CAGGTGGCTGTGCAGGTGAT			
S028	*Loc790226*	F: CCATGACTCCGTAGACAAGA	483	Exon	3O16;9 G2;9 G3;9 H8; 10 N2;15B13;26D1
		R: ACTGCCATAGCTACTGCTGC			
S029	*KCTD20*	F: CGATGCAATCACTAAGCTGG	834	ENSBTAT00000027439: Exon 8	None
		R: GCAGTTCTCATCCTTCGCAC			
S030	*RPS4Y1*	F: TGCCAGCCTCTTGTCTCTCT	430	ENSBTAT00000036142: Exon 2	2A3;11 H24;63 N7; 82 N20;97O2;120P24
		R: TACACCTGAGGAGGCCAAGT			
S031	*CDKN1A*	F: GGATCGCTAAGAGCCGGACA	861	ENSBTAT00000011001: Exon 3	None
		R: GGCAGTCGCTGCTTGAGGTA			
S032	*PPIL1*	F: AATGGTCAATGCGCCTGCTT	888	ENSBTAT00000003071: Exon 4	30O17;139 K9;198 M20;271C5
		R: CACCAACGGCAGCCAGTTCT			
S033	*PI16*	F: CCTAGCAACAGAAGCCTCAA	461	ENSBTAT00000002703: Exon 5	54O24
		R: AGGCCAAGATCTCACTGCAA			
S034	*FGD2*	F: CACCTTGGTGACCAACATTC	414	ENSBTAT00000018834: Exon 16	304 K7;318I17
		R: ACTGCCATAGCTACTGCTGC			
S035	*PIM1*	F: AAGCACGTGGAGAAGGACCG	490	UniSTS:463218	None
		R: GACTGTGTCCTTGAGCAGCG			
S036	*TBC1D22B*	F: CTGTCCACCACTCCATGTCT	539	ENSBTAT00000018938: Exon 13	5 K4;26A20;49B1;98 G9
		R: GGACATTCGGACGTGTAACT			
S037	*RNF8*	F: TCTGAATGGTGTCTGGCTGA	708	ENSBTAT00000010959: Exon 3	None
		R: TTCTCGAGCTGCTCCACTCT			
S038	*Loc509620*	F: AGTGGCACACCGAAGCTC	666	UniSTS:267349	25P1;103D16;207 L11; 271 M7
		R: AACTTCCTCTTGAAGCTTTTGC			
S039	*C23H6orf129*	F: GGCAAGAGAACCGCAAGAAC	281	ENSBTAT00000016009: Exon 4	25P1;103D16
		R: GCACGAAGTCCTTCTGGAGC			
S040	*MDGA1*	F: TCTTGGCGTTGCAGAGATGA	228	ENSBTAT00000047505: Exon 16	None
		R: TGTGCGTGTGTCGAACAACC			
S041	*ZFAND3*	F: CGATTGGTTTAATTTTTTTTTTCA	200	UniSTS:34520	159 K21;185 L24;235B3
		R: TGTGAAGTTTGTTAAATGTAAGGAA			
S042	*BTBD9*	F: GATAGGTCTTACGCTGTTAG	155	UniSTS:279369	None
		R: GAATGTACAGAATAGAAGTG			
S043	*Loc781915*	F: AACCTCAAGTGCCTCTCCAG	714	Exon	67D11;70 N21;76E1; 240 K15;240O16
		R: AACAAGTGTAGCCAGCCATC			
S044	*GL01*	F: GATAGGTCTTACGCTGTTAG	155	UniSTS:279369	None
		R: GAATGTACAGAATAGAAGTG			
S045	*Loc525414*	F: GAAGAAGAGGTGATCGGTGTAGAG	216	UniSTS:476833	8 J2;13E21;24 K16; 24 N15;28 L5;112 N3
		R: TTTCTCCTTCCCATACATTTCTGTG			
S045b	*GLP1R*	F: CGAGTGTGAGGATTCCAAGC	418	Exon 4, 5 and intron	80 G15;138P3
		R: GTAGCCCACCGTGTAGATGA			
S046	*C23H6orf64*	F: GTCACAGCCACCATGGAGTC	415	ENSBTAT00000001425: Exon 2	19 F4;80 G15;138P3; 156B12; 336 L24
		R: CGCAAGCTGTTCTCAGTCAA			
S047	*KCNK5*	F: CTCCGACTCTGTGCTGGTGA	774	ENSBTAT00000014756: Exon 5	None
		R: TACCACGCCTTGTACCGCTA			
S048	*KCNK17*	F: AGAGTCCAGGCTCCTTCTAT	493	ENSBTAT00000013646: Exon 5	None
		R: CTGCTATCCTCAGAGTTCCA			
S049	*Loc100139627*	F: GTGGAGGGAACCTGCGGCAC	344	NC_007324.3: designed online	3 L3;51O8;189 L22; 253I5; 270 L14
		R: AGGCCTCGGAAGAGCCCTGG			
S050	*Loc100138924*	F: CTTGGTCTTGCGGGCCCCTG	493	NC_007324.3: designed online	145 G9;146 H11
		R: CCAGGCTCTAGCCCTGCCCA			
S051	*DAAM2*	F: CAGGGAGTGCTCTCAAAGGTAAAGG	307	UniSTS:476834	None
		R: TCCTCCAGCCTGACTTCTCCTTC			
S052	*MOCS1*	F: GGTCCAGGAAGGCTGAAGTG	661	ENSBTAT00000013792: Exon 11	None
		R: GAAGGACGGATGGCTATGGT			
S053	*LRFN2*	F: TTGTCATACACGGCGGTCCT	493	ENSBTAT00000023907: Exon 1	77E2;220 J8;325 J12; 325 J13
		R: AGCTGAGCCTCGACCACAAC			
S054	*UNC5CL*	F: TGACCAACGAGCAGCCACAC	278	UniSTS:476835	None
		R: GCAGCAGGAGGAGCCAGAAG			
S055	*NFYA*	F: GCCGATGAAGAAGCTATGAC	550	ENSBTAT00000013080: Exon 10	76 K24;118P22;136B19
		R: CATGAGATGGAGCTTCCTTG			
S056	*TREM2*	F: ACAACTCCTTGAAGCACTGG	229	ENSBTAT00000009568: Exon 2	86A4;178 L4;208 M19; 282 F4
		R: TGGAGGCTCTGGCACTGGTA			
S057	*TREM1*	F: CATCATTCCTGCAGCATGTG	515	ENSBTAT00000023397: Exon 4	30C8;73 K17;75A11; 75I21
		R: GGCTGTGCCAGGTCTTAGTT			
S058	*LOC783024*	F: CTGAGGACCAAGGCCATGCT	216	Exon	None
		R: TGGTGTGGCACTGCAGGAAG			
S059	*FOXP4*	F: AATTATCGCTCCAAGAGATTCCAC	250	UniSTS:384935	112I1;144 K17;181 F9; 299P14;314 F18
		R: CCCATCCTTGTCTCCTCTTTACAT			
S060	*MDFI*	F: GCTGTGTCCACTGCATCTTG	256	ENSBTAT00000025763: Exon 4	70B14;166C6;181 J11; 202B12; 229A10
		R: GGTCAGGAGGAGAAGCAGAG			
S061	*PGC*	F: GAAATTCTCTGCTAAACCCCTTCA	268	UniSTS:385581	14 G18;24O7;24O10; 103 G9; 139 N14
		R: TCATCTAAGCAGAAACACCAGTAAATG			
S062	*USP49*	F: GATGGAGTTCATGTAGCAGGTGTT	260	UniSTS:385828	None
		R: GGAGCGCAAGAAGGAGGAG			
S063	*BYSL*	F: TCAGAGGACCTGGAAGTGGA	538	ENSBTAT00000013326: Exon 7	3 M12;98 J10;182 F10
		R: CTCTCATGCACAGCAGTGGA			
S064	*TAF8*	F: TGGAGGAAGGAACTTGGTCACAGAG	228	UniSTS:476836	103 M11;133 J10;146 L22
		R: GGTGCTTGAGGTTCGTTGAGTTGAG			
S065	*MGC137036*	F: GAAGCAGGACCGTGAGCAGA	238	ENSBTAT00000017035: Exon 2	100O15;117E7;133 J9; 146 L22;171 L22;176P6
		R: CTACGAGCGCCACAAGACCA			
S066	*TRERF1*	F: GTGTGTCTGTTGCTGCGGTG	643	ENSBTAT00000020376: Exon 1	1O22;17 J12;79 H15; 81 J21;100O15;259 L15
		R: TGGTCTAGGCTTGGCTGTTG			
S067	*Loc786000*	F: TGGCAAGATGGCGGTGCCAG	379	NC_007324.3: designed online	6P21;32P14;142C8; 162E5;195C23;227D22
		R: AGCAGCCTTGGCCCCACTCT			
S068	*UBR2*	F: CTGCAAGCAACTGACCTCAC	169	ENSBTAT00000007833: Exon 2	6P21;129B6;162E5; 163E23;177 M6
		R: CCAACTCAGGATCTTCACCA			
S069	*PRPH2*	F: GTAGTGGACTCCAGGAACTTCG	232	UniSTS:279013	26 J6;26 L8;29 M14; 127A7;134B12;177A2
		R: ACCACAGAGTCACCTGCTGAGA			
S070	*Loc540169*	F: ATGAAAGGGTCAGGCGAAC	130	UniSTS:94727	144A13;164 L3;164 M2;164 M3;172O18;185 N10
		R: ACAGAGCCGCTAACCGTG			
S071	*CNPY3*	F: GAACAGTGGTCTGGCAAGAA	214	ENSBTAT00000021132: Exon 10	98 J16;172O18;185 N10;189O8;289 J21
		R: GTTAGGCTCAGAGCTCGTCA			
S072	*CUL7*	F: TTTCGACCTCGCTCTGAGTT	1,000	UniSTS:270008	74C2;189O8;289 J21; 325 K12
		R: CTCCAGCATGTGCCAGTG			
S073	*PTK7*	F: GACTCAGGAGCCTTCCAGTG	531	UniSTS:268417	54A6;127D14;142 L8; 163O23;204P7
		R: CTGTATTGCAGCTTCCGAGG			
S074	*Loc540077*	F: CTGAATACCTGATCCGATGG	417	Exon	54A6;142 L8;163O23; 204P7
		R: GCATGTGCATGAGTAGGTCC			
S075	*Loc786439*	F: GGCGTCTTTAATCAGGATTTGG	200	UniSTS:222501	None
		R: AATCCAACACTTGAAACCGACA			
S076	*ZNF318*	F: CTGTCTTCACTCGAAGCTCC	438	ENSBTAT00000013481: Exon 1	24 L23;66 G8;83 N5; 119 J9;162 F10
		R: AGCTCCTACTTCGTTCCTCC			
S077	*TJAP1*	F: GAGGACGAGGAAGAGCTGAA	654	ENSBTAT00000035977: Exon 12	None
		R: CGTGCAGAGGATTGAAGGAG			
S078	*POLH*	F: GACAGCCACACACATAAGCA	497	ENSBTAT00000007900: Exon 11	68 F17;71 H18;74P6; 124 L6;250 J4
		R: GTCTCACAGAGTCGGACACG			
S079	*MRPS18A*	F: AGTCGTGAGACCACTGCAGC	191	ENSBTAT00000056429: Exon 6	115P10;176 M14; 233 H10;278 K6;291I13
		R: AGGACCTCCTGAGAGCCTGA			
S080	*VEGFA*	F: GATCATGCGGATCAAACCTCACC	326	UniSTS:471318	12B17;12 H11;30 L7; 63B18;124 J8;249D14
		R: CCTCCGGACCCAAAGTGCTC			
S081	*MRPL14*	F: TCAGAACTGCTCCATTCACG	182	UniSTS:64809	117 J15
		R: CAACAACGTGGTCCTCATTG			
S082	*SLC29A1*	F: GGTGGTCTTTGAGCACGACT	537	UniSTS:207086	None
		R: CCGGAACAGGAAGGAGAAG			
S083	*AARS2*	F: CACTGGAAGCACTGCTGACC	325	ENSBTAT00000018232: Exon 22	None
		R: GCAGCCAGAACAGCCATGTA			
S084	*CDC5L*	F: CCAACTCAGTGGAGGACCAT	750	UniSTS:267825	134E15;147I12
		R: GGCTTTGTTTCTGGATTTGG			
S085	*SUPT3H*	F: CTTCTGCCTGGAACTTGCACTTG	208	UniSTS:476839	23P23;80P15;110 F4;5;6
		R: TGCTTACTGTCTCCCACCTAGATTG			
S086	*Loc536911*	F: TACCAGCCACCGAGACCAA	309	UniSTS:280406	9 G19;9 H22;9I23;24; 59B8
		R: AGAGGCTGTTTGACGCCATAG			
S086b	*CLIC5(BM1258)*	F: GTATGTATTTTTCCCACCCTGC	158	UniSTS:56663	291I15
		R: GAGTCAGACATGACTGAGCCTG			
S087	*ENPP4*	F: GAACCAGCTCACCAATGTGT	595	ENSBTAT00000004547: Exon 2	72 M13;74O6;127 F7; 182 K12;299 N7
		R: TCCTCTGCTTCACCACCTAA			
S088	*RCAN2*	F: TCTTTACTGTCTGAGCCACC	132	UniSTS:69107	None
		R: TACACTCAGAGCTAGTTTGC			
S089	*CYP39A1*	F: AGGTGATGGTGGCAACTATG	200	UniSTS:15671	57E15;181B7;202D23; 213A17;261 M4
		R: CATGTGTCCATAATTTGATTGC			
S090	*TDRD6*	F: GAGTTCTTCCACCTGCCGTC	490	ENSBTAT00000013158: Exon 1	114B7;147E14;190 N9; 329 H12;350E16
		R: ATACCTGAGCCATGCTCTCG			
S091	*Loc785478*	F: TACGCCACCTACACACACAC	439	Exon	65 L20;133 M1;211 N8; 233B22;233O14
		R: GACTGGTAGCTCCTGATCTG			
S092	*GPR116*	F: CACATCCAGTGCTTATTCAT	302	ENSBTAT00000035930: Exon 18	291 M9
		R: TAGACAGAGAAGTTGGCTTG			
S093	*GPR110*	F: AGTGGACAGATACCGGCTGC	452	ENSBTAT00000028795: Exon 10	None
		R: AGGTGTGGCCATGTGATGGA			
S094	*TNFRSF21*	F: CAGAGCAGAAGGCACCAAGT	500	ENSBTAT00000047874: Exon 11	118P16;351 H10
		R: ATTGTCTGCCTCCTTGGTCC			
S095	*LOC785024*	F: GGTTGTCAAGCCACTCGAAT	611	Exon	14B7;79 L8;168 N8; 264 L6
		R: CGGAGTATATGGCCAGTGTT			
S096	*LOC512926*	F: AGAGCAGAAGGCACCAAGTC	437	Exon	27A8;290 J19;351 H10
		R: ACGCTCTGCATCTCATCACA			
S097	*CD2AP*	F: TACCACAACACCAACTGCAT	309	UniSTS:278169	1 H10;14A2;75 J19; 114B12;151 J21;166 L22
		R: TTACCGGGATCACAGAAACA			
S098	*GPR115*	F: CACAGTGGTGGCAGCAATAA	490	ENSBTAT00000003815: Exon 5	None
		R: GAATAGAGTGCAATGCCGGT			
S099	*OPN5*	F: CTACATCTGCCTGGCGGTCA	287	ENSBTAT00000021933: Exon 4	167I8;228 M7
		R: CATGGCTGCTATGGATCCGA			
S100	*MGC148542*	F: ACATTTTCTCCTTCTTTGGCTCC	272	UniSTS:133880	1A19;1B9;140A1; 216D18;319I16
		R: GATAGAGGATGACGACAAATGGC			
S101	*Loc785693*	F: AGCCAGGTAGAGTTCCAATG	518	Exon	17 K13;75E1;76B22; 103 F21
		R: AGTCTCGGCAGTTACCTTGA			
S102	*MUT*	F: AGCAAAGCACATGCCAAAAT	750	UniSTS:279392	74 J7;8;86P12;252B10; 255 G2;266O16;313 L2
		R: TTCCCCAGAAGAAAGACAAC			
S103	*Loc787783*	F: GGAATCATCAACCCAGTGAGAAAGC	269	UniSTS:476844	255 G2;266O16;274D6; 288I23
		R: CACACGGCGGCAGAAAGAGG			
S104	*RHAG*	F: GAATCGATGACCATCCATGC	470	ENSBTAT00000015012: Exon 4,5	53D7;173C22;186 L10; 226 G3;4;226 H7
		R: AGAAGGCTGGAACATGCGTA			
S105	*Loc100138627*	F: AATGAATAGTATCCCCAATACCTGC	150	UniSTS:164033	None
		R: GTCCACAAAACATTCTCCTTTCC			
S106	*TFAP2D*	F: TAAGCTTTCGGAGAAACCCA	1422	UniSTS:482175	5 K4; 139 L18;230 K5
		R: CAGCAGCAAGACTCTCTGGA			
S107	*TFAP2B*	F: TGCATGCTCCCTCCTCTC	120	UniSTS:71657	25D11;25 F24;142E22; 161A23;167 J23;189D14
		R: CCTCGTCCAATTATGGTGCT			
S108	*Loc100138859*	F: GGAGCACCACAGTACGTAAG	561	Exon	None
		R: GAGGTGTGCCTGTATTGCTA			
S109	*Loc537895*	F: TTCTCTCAAATGATGAATATGCTTC	270	UniSTS:251053	56 J7;86O3;87 H23; 277 G10;277 H11
		R: GGACTATTCTATGCATGCCTCTC			
S110	*IL17A*	F: CACTCAGGCTGTATCAATGC	591	ENSBTAT00000002786: Exon 3	13B24;74A7;74E17; 164 H22;164I23
		R: CAGCTGTGTCATGTACTCCA			
S111	*MCM3*	F: TGTCCCGATTTGACCTTCTC	515	UniSTS:268664	69 G8;168E20;223C7; 263 M23;270P6
		R: GTCATCAGGGCTGAAGTTGG			
S112	*PAQR8*	F: TCTATGTCCTGTCCTCCATC	447	ENSBTAT00000035844: Exon 2	102 M1;160 L10
		R: AGAAGAAGTAGGCACTGACC			
S113	*TRAM2*	F: TGTTCTACATCTTCATCGCCA	630	UniSTS:267311	13P23;53 J18;92C23
		R: ACCAGATCACCGAGCTGAGA			
S114	*TMEM14A*	F: CTACCCAAGAAACACTGTCGC	286	ENSBTAT00000006857: Exon 6	2C18;31C1;139B24; 183A23;280 K17
		R: AGAGCATTCTATGAAGCCCG			
S115	*ICK*	F: ACGGACTGGATCGCTAAGTA	627	ENSBTAT00000020711: Exon 14	2C18;76A8;77 G6; 198C12;199 K7
		R: CAGAACAGCACAGCGGTATT			
S116	*GCM1*	F: AGCTGTCCAACTGCCTCCTG	363	ENSBTAT00000010709: Exon 6	141A15;199 K7;230E24; 314I2
		R: TGGGAAGGGGAGAAGTCGTA			
S117	*ELOVL5*	F: CTACAGCCACGAGACAGTTT	182	UniSTS:279336	64 N21;82O21;90C20; 127 J19;163 F13
		R: GGTTTCAATCATTCTTTCAT			

^*^ The bovine oligo primers were designed along the target bovine genomic sequence at an interval of ~80-160 kb between the two neighbor loci, depending on the availability of the DNA sequence that meet the primer selection criteria. A total of 119 pairs of primers were listed here.

### BAC library organization and screening

To facilitate large scale PCR screening, all the 190,500 clones of the BAC library were organized into 3-dimensional BAC clone pools of plates, rows, and columns. Random BAC clones from each of 496 permanent 384-well storage plates were duplicated onto a Luria-Bertani (LB) agar plate for overnight growth at 37°C, using a 384-pin Multi-Blot Replicator as tool for BAC clone duplication (V & P Scientific, Inc., San Diego, CA). The overnight *E. coli* colonies were then harvested and pooled for plate (n = 496), row (n = 16), or column (n = 24). The standard alkaline lyses methodology was adapted for isolation of the pooled BAC plasmid DNA and the resulting DNA was assembled into super plates for routine PCR screening [35]. The first dimension of the BAC clone pool consisted of 496 DNA samples, each representing one of 496 BAC plates (P001-P496). The second and third dimension consisted of 16 and 24 DNA samples, respectively, for the pooled 16 rows (R01-R16) and 24 columns (C01-C24) of the random BAC clones.

To screen the BAC library using each of 119 pairs of comparative oligo primer pairs, the diluted DNA from each well of the super pool plates was used as a DNA template. The individual PCR reaction was adapted in a total of 10 μl reaction volume with 50 μM of dNTPs, 1.5 mM Mg^++^, 0.2 μM of each primer pair, 1 × PCR buffer, and 0.1 unit of Tag DNA polymerase. The PCR products were resolved by 1.5% agarose gel electrophoresis and the specific PCR fragment band with the expected size indicated a potential positive BAC clone for the gene loci of oligo primers used. The exact location of the target clone in the BAC library was determined by sequential PCRs using the super row and super column DNA as templates, respectively.

### DNA fingerprinting and contig assembling

DNA fingerprinting was performed to determine the overlapping relationship among the identified positive BAC clones [12]. DNA from the positive BAC clone was purified from host *E. coli* by QIAGEN column and subjected for complete restriction enzyme digestion using *Hin*dIII. The enzyme digested products were analyzed on 1% TAE agarose gel electrophoresis for recoding of DNA fragment patterns. The fingerprinting images were captured with UVP Labworks System (UVP Inc., Upland, CA) for systematic analysis. Restriction fragment patterns were analyzed to identify overlapping BAC clones, which were then manually assembled into draft contigs based on the modified methods of Marra [36] and Soderlund [37].

### BAC-end sequencing

BAC-end sequencing was performed for the selected clones to facilitate verification of the overlapping relationships of the BAC clones. The sequencing was performed on an ABI 3730X DNA analyzer at the core facilities of the Institute of Genetics and Developmental Biology, the Chinese Academy of Sciences. The oligo nucleotide primers used for the DNA sequencing were Copycontrol pCC1BAC vector-derived sequencing primer T7 (5’-TAATACGACTCACTATAGGG3’), pCC1/pEpiFOS RP-2 (abbr. RP2) (5’-TACGCCAAGCTATTTAGGTGAGA-3’), and pCC1/pEpiFOS RP-1(abbr. RP-1) (5'-CTCGTATGTTGTGTGGAATTGTGAGC-3'). The resulting sequences were analyzed for overlapping, and used as templates for oligo primer design. Based on the sequence data generated by BAC-end sequencing, PCR primers (Additional file 1: Table S1) were designed to amplify the common genetic loci in two overlapped BACs for confirmation. Sequence-Specific PCRs (SP-PCRs) were performed in 20 μl system including approximately 2 ng BAC DNA, 0.5 U Taq DNA polymerase, 0.1 mM dNTPs, 1.5 mM Mg^++^, 0.25 μM each primer, and 1× PCR buffer. When necessary, the PCR products were verified by cloning the fragments into a TA vector for verifying DNA sequencing.

### Assemble of the BAC clone contig

A continuous BAC clone contig was eventually assembled based on the integrated results of DNA fingerprinting, BAC-end sequencing, and sequence specific PCR amplification of the common loci on the overlapping clones. Redundant BAC clones were removed from the assembly based on the necessity and the relative contribution of each overlapping BACs on the contig. Gaps in the contig were closed by the repeated cycles of PCR screening of BAC clones, DNA fingerprinting of additional BAC clones identified, BAC-end sequencing, and SP-PCR verification. Additional effort was made to link the existing BAC clone contig to the physical map constructed previously, for a complete physical map covering the entire ovine MHC including the autosome insertion between class IIa and IIb.

For comparison of the MHC structure and organization between sheep and other mammals, multiple comparisons were performed for the representative MHC and extended DNA sequences from human, chimpanzees, mouse, cattle, and sheep. Sequence data were downloaded from the NCBI database and other related public websites designated for the sheep genomic information.

## Results

### Target BAC identification

We successfully identified a total of 368 positive BAC clones for ovine chromosome 20 between MHC class IIa and IIb, utilizing bovine primers designed from the consensus genome region (Table1). Out of 119 pairs of oligo primers designed, 92 pairs worked effectively to generate specific target gene fragments of the expected sizes. This approach resulted in the successful identification of positive ovine BAC clones in the target genome region, and the overall efficiency of comparative PCR reached 80%. The relatively high rate of success for the comparative SP-PCR not only facilitated our mapping efforts, but also helped to confirm the homologous nature of MHC regions between bovine and ovine species.

**Figure 1:**
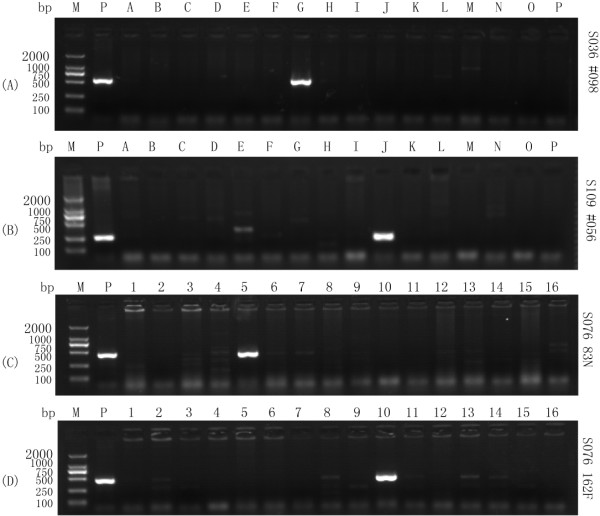
**Representative gel images on initial PCR screening of an ovine BAC library using comparative primers from the bovine sequences.** Approximately 190,500 random BAC clones were organized into pooled super DNA plates of rows, columns, and plates to facilitate PCR screening. Location of a target positive BAC clone in the library was determined usually by two runs of PCRs, one for “plate” and the other for “row + column”. The procedure eliminated the need for hybridization-based screening with radioactive ^32^P labeling. Gel images of PCR screen band on (**A**): Row pool of P098 BAC plate using the primer pair S036; (**B**): Row pool of P056 BAC plate using the primer pair S109; (**C**): Row N of P083 BAC plate; (**D**): Row F of P162 BAC plate. M: DL2000. Sample: PCR Products. A ~ P: Number of Row. 1 ~ 16: Number of Column (only partial shown here). P: Positive control (The amplified PCR products using the sheep genome DNA as templates).

Organization of ~190,500 random ovine BAC clones into three dimensional super DNA pool of rows (n = 16), columns (n = 24), and plates (n = 496) significantly increased the efficiency of PCR screening of the sheep BAC library (Figure1). The whole BAC library of 8.4× genome equivalents was screened through with a maximum of 536 (=496 + 16 + 24) PCR reactions, and a positive BAC clone could be frequently identified by as few as 136 (=96 + 16 + 24) PCR reactions using the super pool DNA as templates. In addition, PCR-based BAC clone screening also helped to eliminate the need for hybridization-based screening using radioactive ^32^P labeling.

### DNA fingerprinting and BAC-end sequencing

**Figure 2:**
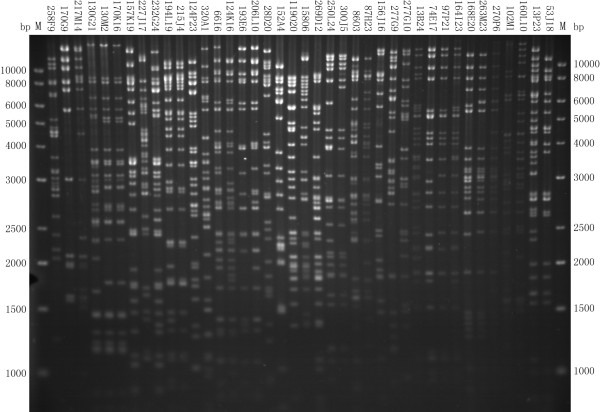
**A representative image of DNA fingerprints of the positive BAC clones for determination of overlapping relationship.** The positive BAC clones identified in the previous steps were digested with *Hin*d III, followed by separation on a 1% agarose gel in 1× TAE buffer. The gel was stained with Ethidium Bromide (EB) for photograph with a UVP Labworks system. M: Marker of DNA size standard (1 kb plus DNA ladder from Invitrogen, San Diego, CA, USA) with the base pair (bp) sizes indicated on both sides.

The initial order of the positive BAC clones identified was successfully determined by inferring the overlapping relationships among the clones via DNA fingerprinting, using *Hin*dIII for restriction enzyme digestion of the BAC clone DNAs (Figure2). Out of 368 positive BAC clones subjected for the DNA fingerprinting, 185 clones with their overlapping relationships were successfully determined. The resulting BAC contig covered the entire autosome insertion region between the MHC class IIa and IIb. After removing the redundant clones, a total of 108 effective BACs were ordered to form an overlapping BAC contig (Additional file 1: Table S1).

For cross-checking of the clone order, BAC-end sequencing was performed for all overlapping BAC clones, and the sequences generated were used to design BAC-end oligo primers (Additional file 1: Table S1) for further verification of overlapping relationships. The sequences of 185 BAC-ends have been deposited into the NCBI database with the access number HR309252 through HR309068, corresponding to dbGSS ID 30164010 through 30163826.

### Cross verification and physical map assembling

**Figure 3:**
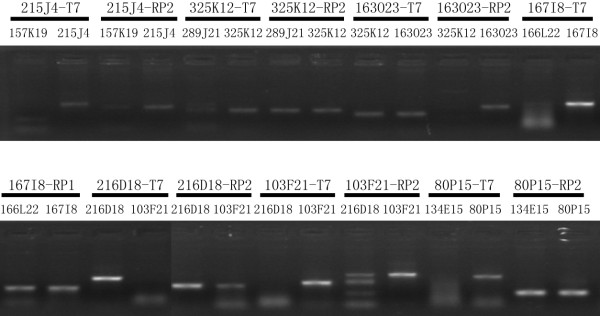
**PCR verification of the overlapping relationship between pairs of overlapping BAC clones.** Pairs of overlapped BAC clones were PCR amplified using a primer pair designed based on the BAC-end sequence. The markers above the black lines define the primer pairs and the ones below the lines are numbers of positive clones used as PCR templates.

For additional cross-verification of the BAC clone orders, a total of 108 pairs of BAC-end oligo primers were designed for amplification by PCR of the common loci in two overlapping BACs (Figure3). Verification PCR confirmed the results of DNA fingerprinting at a high level of accuracy. Out of the 108 primer pairs used, 103 produced the specific PCR products with the expected size, the overall success rate reached 95% (Additional file 1: Table S1). An overlapping relationship between two BACs was further verified if the common target loci were detected from both BACs in the overlapped region. A total of five pairs of oligo primers failed to generate the specific PCR band, or failed to produce the PCR fragment at the expected size.

**Figure 4:**
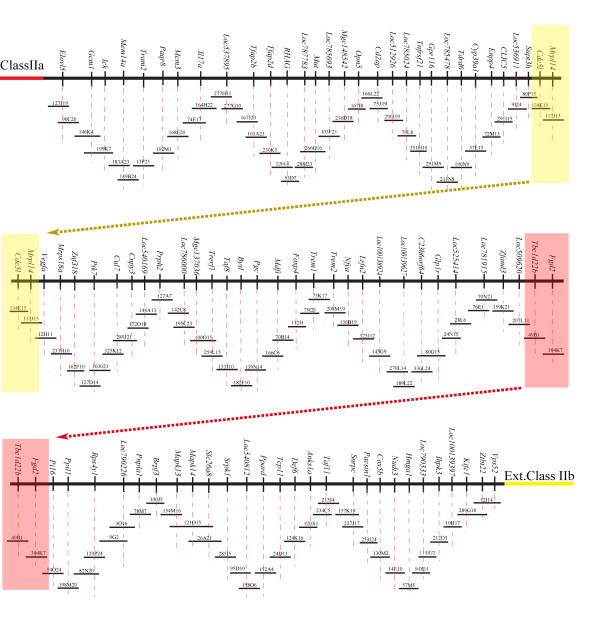
**A 14 Mb BAC clone physical map covering the entire region between ovine MHC Class IIa and IIb.** The order and orientation of BAC clones (overlapping horizontal bars with clone ID name listed above) were determined by combinations of DNA fingerprinting, BAC-end sequencing, and sequence-specific-PCR. Target gene identified by BAC-end sequencing is marked with a vertical bar along the horizontal line, with locus name listed above. The continuous BAC map is represented by three panels with the overlapping regions marked with the same colored shadows at the both ends.

A complete physical map of a BAC clone contig for the ovine MHC region between class IIa and IIb was successfully assembled (Figure4), based on the integrated results of DNA fingerprinting, BAC-end sequencing, and confirmation PCR of the BAC ends. The fully assembled physical map was composed of 108 effective ovine BAC clones organized into a continuous contig that covered the entire region between ovine MHC class IIa and IIb (Figure4). Based on the results of DNA fingerprinting, no gaps exist in the constructed BAC clone physical map which spans approximately 14 Mb genome region of ovine chromosome 20, indicating the even distribution of BAC clones in the library we previously constructed.

## Discussion

**Figure 5:**
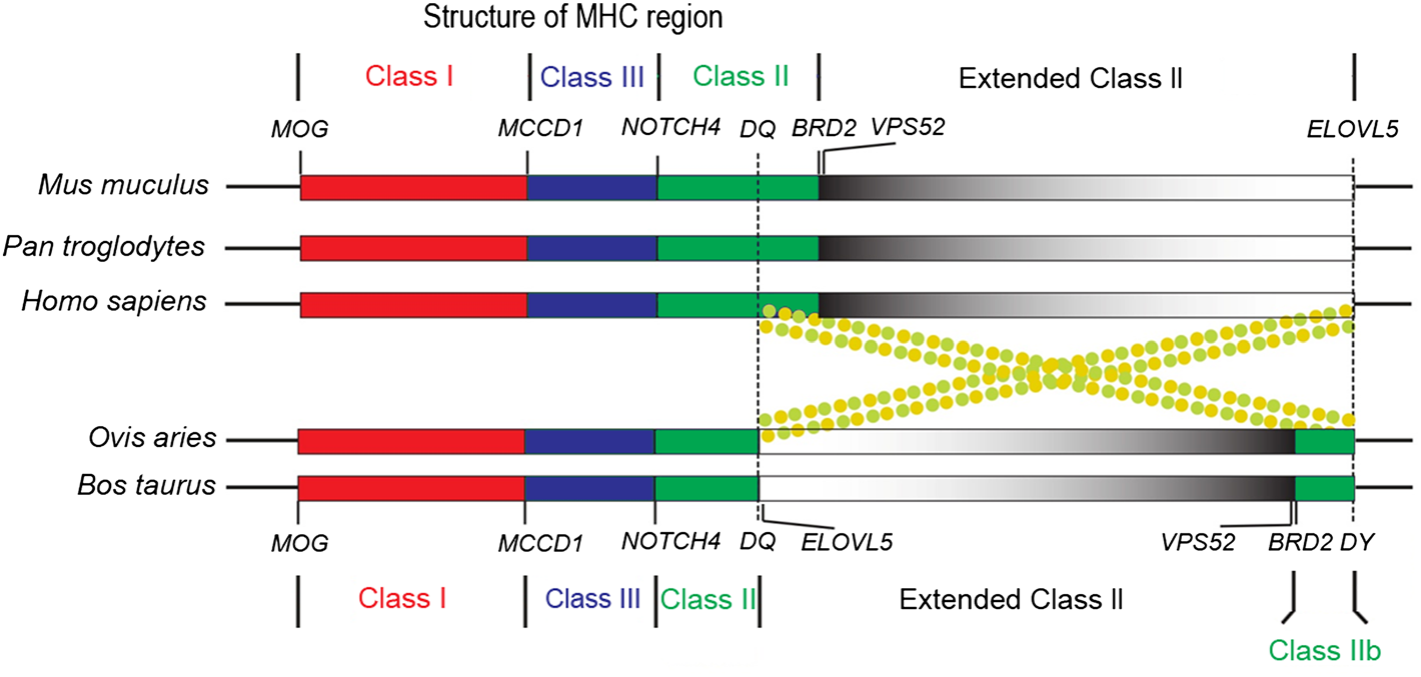
**Schematic presentation of MHC structures among representative mammal species.** Bovine and ovine MHC is interrupted by a long piece of non-MHC insertion that divided class II into IIa and IIb subregions. The red, blue, and green color stands for MHC Class I, Class III, and Class II, respectively. The grey color gradient represents the extended Class II region. The order of loci in the extended Class II region of bovine and ovine is in an opposite orientation compared to that of human, chimpanzees, and mouse. Dash line marks the break point of a hypothetical chromosome inversion. Dashed circles indicate the hypothetical chromosome looping and the subsequent crossover occurred during the evolution of ruminants. The drawing is not to the scale.

Using the comparative approaches, we successfully constructed a 14 Mb BAC clone contig map for a region in ovine chromosome 20 that harbors the MHC. Comparison between the identified ovine BAC contig and the orthologous bovine genomic region showed that the two species share essentially the same genomic structure and organization for the entire inversion/insertion between MHC class IIa and IIb (Figure5). For the available genetic loci generated via the SP-PCR and BAC-end sequencing, our results essentially confirmed the sheep genome sequence assembly presented by ISGC in the MHC region [33].

The physical map of ovine BAC contig we constructed helped to provide additional evidence to support the hypothesis that, there was an ancient chromosome rearrangement in the ancestor of ruminants which shaped the MHC structures currently observed in the ovine and bovine (Figure5). It is obvious that the MHC region in human, mouse and chimpanzees is continuous with no interruption, but in bovine and ovine it is interrupted by a large piece of autosome insertion which divided MHC class II into IIa and IIb subregions (Figure5). Given the fact of opposite loci order and orientation for the insertion region in ovine and bovine relative to those of human and mouse, it is highly possible that an event of genetic recombination occurred to the ancestor chromosome of ruminants, probably via chromosome looping and the subsequent crossover. This possibility was suggested by researchers previously [29,38].

Examination of the bovine DNA sequence from the public database showed that the total length of bovine MHC is ~20 Mb, including the extended Class IIb region [34]. However, the total length of the orthologous ovine MHC was ~14.3 Mb as determined in this study, which is approximately 5.7 Mb shorter than the MHC of bovine. On the other hand, the sequence of the same bovine region presented in the NCBI database is ~18 Mb in length (http://www.ncbi.nlm.nih.gov/projects/mapview/maps.cgi?taxid=9913&amp;chr=23). These discrepancies may not likely be resolved unless highly accurate sequence maps for the entire MHC regions become available.

The reliability of the ovine BAC contig map reported here is sufficiently high in theory, partially due to the fact that the DNA fingerprinting was utilized to infer the BAC clone orders, plus the results were cross-verified by both of the BAC-end sequencing and SP-PCR amplification of the target loci. However, it is not escaped from our attention that there are 5 out of the 108 overlapping locations in the BAC map where the SP-PCR failed to generate the expected PCR products between the overlapping BAC clones (data not shown). The significance of such failure in relation to the overall quality of the map remains to be determined. The possible explanations include the error in SP-PCR primer sequences, the high level of heterogeneity or polymorphism of the target locus involved, or the mistake in the interpretation of results of DNA fingerprinting.

Combined with our previous BAC physical map for the ovine MHC, we have now assembled a completed BAC clone physical map with the inversion/insertion region included (Additional file 2: Figure S1). The physical map will help to generate an ovine MHC sequencing map with a high level of accuracy, which in turn will facilitate MHC functional studies and comparative MHC evolution studies in ruminants. DNA sequencing of the BACs is currently underway.

## Conclusion

We constructed a high-density physical map for the sheep genome region between MHC class IIa and IIb via comparative approaches. A total of 108 effective ovine BAC clones were selected to form a continuous BAC contig that covers the entire non-MHC insertion. The map spans approximately 14 Mb in length, constituting ~25% of ovine chromosome 20. The entire ovine MHC region, including the autosome insertion for which the physical map has been constructed, is now fully covered by a continuous BAC clone contig. The accuracy of DNA sequences play vital roles in detailed SNP and other functional studies of MHC genes, as well as for genome evolution studies. The physical map will help to generate ovine MHC sequencing map with a high level of accuracy, which in turn will facilitate MHC functional studies, as well as the comparative MHC evolution in ruminants.

## Misc

Gang Li and Ka Liu contributed equally.

## Competing interests

Authors declare no conflict of interests.

## Authors’ contributions

GL carried out BAC library organization and SP-PCR screening. KL carried out DNA fingerprinting and contig assembling. SJ and GL performed oligo primer design and BAC-end sequencing. HL constructed the sheep BAC library. HB carried out data analysis. XC carried out certain verification experiments. PT and PZ carried out data cross checking. RM and JG supervised the studies and wrote the manuscript. All authors read and approved the final version of the manuscript.

## Supplementary Material

Additional file 1 **Table S1.** The ovine oligo primers used for verification of overlapping relationships of the positive BAC clones. Click here for file

Additional file 2 **Figure S1.** A complete physical map of entire ovine MHC with the insertion region between class IIa and IIb included. Order and orientation of overlapping BAC clones were jointly determined by combinations of DNA fingerprinting, BAC-end sequencing, and sequence-specific PCR. Genes identified by BAC-end sequencing are marked with erect black lines, with their names listed above. A horizontal bar stands for individual BAC with its identification marked above. Red, purple and green color represent the MHC class I, class III, and class II, representatively. Click here for file
