# Supplementary material for: A physical map of a BAC clone contig covering the entire autosome insertion between ovine MHC Class IIa and IIb
Source: BMC Genomics. 2012 Aug 16;13:398. doi: 10.1186/1471-2164-13-398 (PMC3475007; doi:10.1186/1471-2164-13-398)
Supplement: Additional file 1 — Table S1. The ovine oligo primers used for verification of overlapping relationships of the positive BAC clones. [file 1471-2164-13-398-S1.doc]

**Table S1. The ovine oligo primers used for verification of overlapping relationships of the positive BAC clones**

| **Primer Name** | **DNA Sequences (5'→3')** | **Product (bp)** | **Ann. T.**  **(°C)** | **BAC Clones Verified** |
| --- | --- | --- | --- | --- |
| 12J14-T7 | F: TCCATCACCTCCACTAGCTT  R: TGACCACCTGTGAGGCTAAT | 290 | 55 | 12I12;12J14;  289G18 |
| 12J14-RP2 | F: AGCTCTCGTGACTGAGGTTA  R: GTATGTCTGCTCTTGCTGCT | 356 | 55 | 12J14;12I9;  58G13 |
| 12I12-T7 | F: TGATTCCTTGACCGTGCCTG  R: AGCGAGAGCTTCACCGAACA | 176 | 62 | 12I12;12J14;  289G18 |
| 12I12-RP2 | F: GGAGACCTGCTTCTAAGCCT  R: CTACCTTCTGACAGCCATGC | 324 | 58 | 12I12;12I9 |
| 289G18-T7 | F: CCTCTCTGGTCTTGCAGGTT  R: AGTGCATGGACTCAGAGTGG | 499 | 60 | 289G18;19H17 |
| 289G18-RP2 | F: CAGTGAGCAACAGCCTCTTA  R: GCTCACATGGTGTGTCTGTC | 208 | 55 | 289G18;12J14 |
| 19H17-T7 | F: TCATCAGTGGCCTGACTAGC  R: CATGTCCTCTGGATGCCTTA | 373 | 60 | 19H17;212D3 |
| 19H17-RP2 | F: ACACTGTGGTAGGTGCCTTA  R: CTTTGTTTGGTAGCCACACT | 355 | 55 | 19H17; 289G18 |
| 130M2-T7 | F: GGCAAGGTATTCAGTGAGGA  R: GGAGACTGGTTGCACCTAGA | 464 | 58 | 130M2;253I24 |
| 130M2-RP2 | F: CTGGCAAGGTATTCAGTGAG  R: AAGGAGACTGGTTGCACCTA | 468 | 55 | 130M2;14E10 |
| 227J17-T7 | F: TTCACAAGGCAGGAGAGATG  R: ACAGTGAAGTCGCTCAGTCG | 489 | 60 | 227J17;157K19 |
| 227J17-RP2 | F: TGTCATGAACCTCCATCCAG  R: GACATCACCAGATGGCCAAC | 288 | 60 | 227J17;253I24 |
| 223N7-T7 | F: TTCACAAGGCAGGAGAGATG  R: CAGTGAAGTCGCTCAGTCGT | 488 | 60 | 227J17;157K19 |
| 223N7-RP2 | F: CTTGCTGGCTACTTCCTGTT  R: CCAAGGAGAGAGGAGGAGAT | 449 | 58 | 227J17;253I24 |
| 157K19-T7 | F: CCAACTTGCCGTGTGTGTAT  R: CCTCTCCTTATGGCACTGAA | 499 | 60 | 157K19; 227J17 |
| 157K19-RP2 | F: ACTTCAGCATCAGTCCTTCC  R: ACTCTGCTCACAAGTGTCCA | 228 | 55 | 157K19;215J4 |
| 215J4-T7 | F: TTCCAGCTTGTGCTTCATCC  R: CACCACTGAGCAACTTCACG | 426 | 60 | 215J4;320A1;  124P23 |
| 215J4-RP2 | F: GAGACATCACCTTGAGACCA  R: GTCTTACCCTCTGTTGTCCC | 345 | 55 | 215J4; 157K19 |
| 234C5-T7 | F: GTACAGTACAATGGCTGGCA  R: CTGAATTGAGTCCAGCAAGG | 199 | 55 | 234C5;320A1;  124P23 |
| 234C5-RP2 | F: GACACAACTGAGCAACCAAC  R: CTCCTCTCTAAGCAATCCTG | 286 | 55 | 234C5; 215J4 |
| 320A1-T7 | F: AGGACACGGCTGGCAATAGT  R: GGCGGTAGAATGGAACAGGA | 360 | 62 | 320A1;124P23;  234C5 |
| 320A1-RP2 | F: AGAGCATGTGGTCTTCCTGG  R: GCAGGACTGAGGAAGAAGCA | 268 | 60 | 320A1;124P23;  124K16 |
| 124K16-T7 | F: CACGACCAGATGCAGTGACA  R: CCACCTGGTAGAAGCAGCAA | 441 | 60 | 124K16;66I6;  24D11 |
| 124K16-RP2 | F: ATAGCTTGGCGTAAGGTCTC  R: TTCAGTGGTTAGGACTCTGC | 486 | 55 | 124K16;66I6;  320A1 |
| 24D11-T7 | F: GCAGAAGACAAGAGATGGCA  R: CCACCGATGGCACTACTCTA | 277 | 60 | 24D11;28D20;  152A4 |
| 24D11-RP1 | F: CCAATCCACAGAACAACAGG  R: AGCAAGCAGCAAGGAACACT | 293 | 60 | 24D11;124K16 |
| 152A4-T7 | F: TGGACAGAGAAGCCTGGTGG  R: AGCTCTTCGTGTGTGCCTGC | 288 | 62 | 152A4;24D11 |
| 152A4-RP2 | F: CCTGGTAGCTCAGTTGGTAA  R: CCAGACGCTATCCTCAGTAA | 387 | 55 | 152A4;158O6 |
| 158O6-T7 | F: TCCTAGAGTCGACACTGCAT  R: CCAAGACAGAGCTGTAGGAT | 380 | 55 | 158O6;152A4;  28D20 |
| 158O6-RP2 | F: AGACTGCCAGCTGAGCCATA  R: TTAACCAACAACTGCAGCCC | 421 | 60 | 158O6;95D10 |
| 95D10-T7 | F: CAAGTGCTGGTGAAGATGGA  R: GTATAATGCCCTTGGGATGC | 371 | 60 | 95D10;158O6;  119O20 |
| 95D10-RP2 | F: GTTAGGAACGAGTTGCTGGT  R: TGGTGGCTGTCATCTGTTAG | 262 | 55 | 95D10;285I5;  269D12 |
| 285I5-T7 | F: GAACCGTGTGCTCTGTATCT  R: ACTCTGTGTGCTCATGTTCC | 200 | 55 | 285I5;26A21 |
| 285I5-RP2 | F: CATTGGCCTGGATCACTCAC  R: CACTACCGTCAGCTCAACGA | 230 | 60 | 285I5;95D10;  269D12 |
| 300J5-T7 | F: TTGAGGCCACCAGAAGCTAA  R: ACGGCTGCTAATCGAACTGA | 223 | 60 | 300J5;154M16 |
| 300J5-RP1 | F: TCTCATGACCACTGTGACCA  R: CCACTCCAGTATTCTTGCCT | 353 | 55 | 300J5;78M7;  153F9 |
| 78M7-T7 | F: TTCGTCCAGAAGGTCATTGC  R: CCTGGACTTGAAGTTGCTGC | 378 | 60 | 78M7;300J5 |
| 78M7-RP2 | F: CCTGGATGTGCTGAAGCTGG  R: CAACTGAGCACAGAGGCGGA | 224 | 62 | 78M7;3O16;  15B13 |
| 9G2-T7 | F: CATCCTCATTCTGCACTCAC  R: GCCAGAAGAAGAGAGGAGAC | 340 | 55 | 9G2;120P24;2A3 |
| 9G2-RP2 | F: CTTGTCCAAGGTCACATAGC  R: TGATCTCGTCTTCATACCCA | 599 | 55 | 9G2;3O16;  15B13 |
| 82N20-T7 | F: TCATTCAGCCTCCAAGACCT  R: GCATCATCAGCCTTCTGGTT | 277 | 60 | 82N20; 2A3;120P24 |
| 82N20-RP2 | F: CATGTGAGACTATGCCACCT  R: CCAGTGGTCCAAGTCCTTAT | 452 | 55 | 82N20;198M20 |
| 198M20-T7 | F: TCTGCAGTCTGTGGTGTTCA  R: CTCGCAGACAACCATTCATC | 213 | 60 | 198M20;82N20 |
| 198M20-RP2 | F: CCATGTATTCTTGCCACCTC  R: TGGCTCAGATCATGAACTCC | 558 | 58 | 198M20;54O24 |
| 54O24-T7 | F: TGCCTGGTGTAGAAGCTGTA  R: CACTGTGCTAGGAGGTGTGA | 486 | 55 | 54O24; 304K7;318I17 |
| 54O24-RP2 | F: TAGCCATCATGGTCAGCAAA  R: CCTGCTTCATTCCACATTCC | 305 | 60 | 54O24;198M20 |
| 304K7-T7 | F: AAGAGCCTGTCTCCTTGTGC  R: TCATGGCCAACACTCAGGTA | 280 | 60 | 304K7;54O24 |
| 304K7-RP2 | F: CCATTGAGTCAGTGATGCCA  R: CTGCCATCCTGAGGAGAAGA | 398 | 60 | 304K7;49B1 |
| 49B1-T7 | F: CCACATTGGCAGGTGAGTTC  R: TTCCAGCTTGTGCTTCATCC | 418 | 60 | 49B1;304K7;  318I17 |
| 49B1-RP2 | F: TGTGCCAGCCTCTTCACTTA  R: AGTGTTATCTGCCTGCAACG | 297 | 60 | 49B1;207L11 |
| 207L11-T7 | F: TTGTATCCATCTCTGCCACC  R: TTCAACCAGAGGAGCAGAAC | 367 | 58 | 207L11;49B1;  5K4 |
| 207L11-RP2 | F: AACTTCATGGAGACTCCAGG  R: AGGAAGTGTCAGGTGTCAGC | 340 | 55 | 207L11;103D16;  159K21 |
| 159K21-T7 | F: TCATTAGCAGGCAAGTCTGG  R: GCCTTCATCTTGCATGTCAC | 490 | 60 | 159K21;70N21 |
| 159K21-RP2 | F: AAGAAGGTCCAACTCTGGCA  R: TCCACAGCAATGTCTCCTGA | 256 | 60 | 159K21; 207L11;103D16 |
| 70N21-T7 | F: GTTGTTCCATCTCCAGCTCT  R: CACAATGGCACTCATCTCAC | 348 | 55 | 70N21;76E1 |
| 70N21-RP2 | F: GCCTAAGCTGAGATAGATGG  R: GGAGTCCTGCTTCAGTAATG | 320 | 55 | 70N21;159K21 |
| 76E1-T7 | F: AGCCAGATTGTCTGAACGTC  R: GCATAGTCAGGCAGGTTCTT | 343 | 55 | 76E1;70N21;  240K15 |
| 76E1-RP2 | F: AACTCACTGCTGCTGCTGCT  R: AATGTCGCTCAGTCGTGTCC | 467 | 60 | 76E1;28L6 |
| 24N15-T7 | F: ATGTGCAGCTGTACGAGTCC  R: TCTCCTGCAAGGCACATAGA | 306 | 60 | 24N15;28L6 |
| 24N15-RP2 | F: CCTGTCAAGCTGAATGTGGA  R: CCCAAGACCTAGAAACACGA | 247 | 60 | 24N15;80G15;  138P3 |
| 336L24-T7 | F: AGAGCTGAGTGCCGAAGAAT  R: CAGTTCAGTCGCTCAGTCGT | 388 | 60 | 336L24; 80G15;138P3 |
| 336L24-RP2 | F: CCACAGGAAGCTGATACCGA  R: GCCTTGTCCTTGTGACTTGG | 472 | 60 | 336L24;189L22 |
| 189L22-T7 | F: ACATGCTTAGGAGGACCTGC  R: GCCAGTTCAGGAGACAGGAG | 346 | 60 | 189L22;336L24 |
| 189L22-RP2 | F: CTTCACGCAGTCAGTCCTGG  R: TGGCAAGTCAGAGCGAAGTC | 546 | 60 | 189L22;270L13 |
| 325J12-T7 | F: TGGTGGTCCAGTGGTTAAGA  R: GTAATGCCTCCTGTGACCGT | 450 | 60 | 325J12;136B19;  118P22 |
| 325J12-RP2 | F: TGATCTGTTTATGAGCCCGT  R: CACACTGGCAACATTCAGGA | 370 | 60 | 325J12;145G9;  146H11 |
| 136B19-T7 | F: TCACATGAACCACAGCCTTG  R: CTCCTCTTCACTTCCTGCCA | 276 | 60 | 136B19;325J12 |
| 136B19-RP2 | F: TTGGACTGAACGCATCTCTG  R: CAGGAACCTCTAGCGTTGTG | 414 | 60 | 136B19;208M19;  282F4 |
| 208M19-T7 | F: GCATGCAAGCTTAGAGAAGG  R: TGACAGGCTACAATGCATGG | 479 | 60 | 208M19;136B19 |
| 208M19-RP2 | F: AGTGAGGTCTCCAGGTGAGG  R: TTGGCTGCATCAGGTCTTAG | 279 | 60 | 208M19;73K17 |
| 73K17-T7 | F: AGATGGACAGCTATGCAAGG  R: ACACAAGAGATGCAGGTTCG | 416 | 60 | 73K17;75I21 |
| 73K17-RP2 | F: GCAGAGAAATACTCAGCAGG  R: CCAGAAGTCTCTGAGGTTGT | 395 | 55 | 73K17; 208M19;282F4 |
| 75I21-T7 | F: AACACCAGCACTTCAGCACT  R: TTCCTGGACATGCACAGAAC | 299 | 60 | 75I21;112I1 |
| 75I21-RP2 | F: GGCAGGTAACAGAAGTGGTA  R: CTGAAGGGAAGCAGTGTAGT | 282 | 55 | 75I21;73K17 |
| 112I1-T7 | F: GGAGAGACCTCAGGTTGAGC  R: TTAGCAGGAGTGGAGAATGG | 204 | 60 | 112I1;70B14 |
| 112I1-RP2 | F: TACGTGTCACCATCCACAGG  R: GTGTCCGAATCTTCGAGACC | 295 | 60 | 112I1;75I21 |
| 70B14-T7 | F: TGCGACCAGAATCATGTACG  R: CCATCTCCATCACATGCAAG | 244 | 60 | 70B14;112I1 |
| 70B14-RP2 | F: GCAATGGACAAGAAGGAAGG  R: CATCAGGAACTCAGGCAGTG | 311 | 60 | 70B14;166C6 |
| 166C6-T7 | F: ACACACACACACACACACCA  R: GAACAGGATTGCTACAGGTC | 360 | 55 | 166C6;139N14;  103G9 |
| 166C6-RP2 | F: AACTGGATTGCTGGATGGAG  R: TCCTTGGCCCACTTTTAGAA | 397 | 60 | 166C6;70B14 |
| 139N14-T7 | F: GCAGTGGAAGCGCAGAGTAT  R: AAGGACAGGAGCTGGTAAGG | 330 | 60 | 139N14;166C6 |
| 139N14-RP1 | F: CCTTCCTGGCTTCCAGACTC  R: GAGGAATGCTGCCATGGTAA | 496 | 60 | 139N14;103G9;  182F10 |
| 182F10-T7 | F: TTGCCAGCACAGCTATCTCT  R: TGCATTGACAGGTGGACTCT | 479 | 60 | 182F10;139N14 |
| 182F10-RP1 | F: TTAGATCTGCGTGGAAGTGG  R: TCGTGTCTGACTCTGTGCAA | 375 | 60 | 182F10;133J10 |
| 133J10-T7 | F: GTCCACTGACCATGCTATTC  R: ATAATTCCAACATCAGCCAC | 220 | 55 | 133J10;259L15 |
| 133J10-RP2 | F: CTCCAAGACTCCAGGCTCAC  R: CACAGTCACCTAGCAAGGCA | 262 | 60 | 133J10;182F10 |
| 100O15-T7 | F: CCACAAGCTGTGCAGTAGAT  R: CTGAGTAAGGCAAGAAGACG | 370 | 55 | 100O15;195C23 |
| 100O15-RP1 | F: ATGTGAGAAGGTGCTCTGGT  R: TGACAGGCGTCTACAGTAGG | 290 | 55 | 100O15;259L15 |
| 259L15-T7 | F: CCAGCCAGGAATGAGTTCTT  R: AAGACGTCGTCCAGCAGTTA | 405 | 60 | 259L15;100O15 |
| 259L15-RP2 | F: AATACTAGGTCCGAGGCATC  R: ACCAATGTCAGTGACCACAG | 191 | 55 | 259L15;133J10 |
| 195C23-T7 | F: GCTCACTTGAGTCAGGCTGG  R: TGGCTTCCTTGATATGCTGG | 411 | 60 | 195C23;100O15 |
| 195C23-RP1 | F: TCTCTAGCTGTGGTGTGTGG  R: GGTCGCACAGAGTTGGATAC | 271 | 55 | 195C23;142C8;  32P14 |
| 142C8-T7 | F: CCTTGAAGGGTTTTAAGCTG  R: TCATAGGTCCTTTCTTGCCA | 429 | 58 | 142C8;127A7 |
| 142C8-RP1 | F: CTCAAGCTTCACATCACTGC  R: GACTTCAAGATTGAGCCTGG | 479 | 55 | 142C8;195C23 |
| 127A7-T7 | F: ATGTCCGTCGAGTTGGTGAT  R: TGTGGCGAACTATGGTGTTG | 262 | 60 | 127A7;142C8;  6P21;162E5 |
| 127A7-RP2 | F: TGCCAGGTCTTGTGTTAAGG  R: CAACTCAGAAGCCATCATGC | 561 | 60 | 127A7;144A13 |
| 144A13-T7 | F: CAGTCACAGGAGATGAGGCA  R: TGCTTGGAACTCAGACTTGG | 246 | 60 | 144A13;127A7 |
| 144A13-RP2 | F: CAGGAGGAATGCAGGTTAGG  R: AGCCGATTACAGACCACCAG | 427 | 60 | 144A13;172O18;  185N10 |
| 172O18-T7 | F: ATTGCTCTGGCTAGGACTTG  R: GCTCAGTCGCTAAGTCGTGT | 344 | 58 | 172O18;185N10; 289J21 |
| 172O18-RP1 | F: CCATGATTACGCCAAGCTAT  R: CCTTACAGAAGCCTCACTCC | 609 | 55 | 172O18;185N10; 144A13 |
| 289J21-T7 | F: GACACGACTGAAGCGACTTA  R: TGCTGAGCTTGGTAGGATAG | 189 | 55 | 289J21; 172O18;185N10 |
| 289J21-RP2 | F: CCTTCTTCAGCGATCAATGC  R: AAGGCCCACTTGACTTCACA | 286 | 60 | 289J21;325K12 |
| 325K12-T7 | F: TTGACTCACCTGGCTATGGA  R: GTGGAATCAGAGCAGACCGT | 274 | 60 | 325K12;163O23 |
| 325K12-RP2 | F: CACTTGCAGCTCTGACTTTT  R: TCAGAACCAGCTCAGAAGTG | 276 | 55 | 325K12;289J21 |
| 163O23-T7 | F: GTGCTGCCACATTCCAGACT  R: GACTTGCCTCCAACGTCAGA | 209 | 60 | 163O23; 325K12 |
| 163O23-RP2 | F: CTACCTGCCGCCTCTACATT  R: AACAGTGTTCTGCCAGGACC | 334 | 60 | 163O23;127D14;  204P7 |
| 127D14-T7 | F: CCACCAGCATTACCACCATT  R: GACGATGCCAGCAAGAAGTT | 253 | 60 | 127D14; 163O23; 204P7 |
| 127D14-RP2 | F: AGGAACCTGGCACTCGATCT  R: GCAACATTGGCAGCTCTGAA | 248 | 60 | 127D14;162F10 |
| 162F10-T7 | F: AGTAGGCAGTCCGAGTAGGT  R: TGGACTCACAGTCAGGAAGA | 452 | 55 | 162F10; 127D14 |
| 162F10-RP2 | F: CCTGGACCTACTCATGCACA  R: GCTATCATTCTACCCCGCTT | 282 | 60 | 162F10;233H10 |
| 233H10-T7 | F: CACTAGGTGTCGCTCCGTTC  R: AGTGACGGAGGCTGCTGATA | 191 | 60 | 233H10;12H11;  124J8 |
| 233H10-RP2 | F: AAGCTGCGCTGGTAGACATC  R: TCTTCATCAGTGTTGGCGGT | 234 | 60 | 233H10;162F10 |
| 12H11-T7 | F: CAGTCAGTGCTGCCTCCTT  R: GTTGGATGAATTGTTCAGGG | 350 | 58 | 12H11;124J8; 233H10 |
| 117J15-T7 | F: CTCGTGGCTCAGTGGTTAAG  R: CTTCTCGTTGTGGAGCACAG | 212 | 60 | 117J15;12H11 |
| 117J15-RP1 | F: TCATGCGGTAGATACGGTTG  R: CATTGTGACCAGCACCTACG | 281 | 60 | 117J15;134E15;  147I12 |
| 134E15-T7 | F: CATCCTGAGCCTGTTCACAA  R: GCCTGTCTCTTCTGCTTCCT | 483 | 60 | 134E15;147I12 |
| 134E15-RP2 | F: AGGCAGTTCTGAGCTAATGG  R: AAGTGAACGATGGTGCTGTG | 256 | 60 | 134E15;80P15 |
| 80P15-T7 | F: GATTCTTTGTGCAATTTGGT  R: CGACTGAGTGATGGAACAAC | 498 | 55 | 80P15;9I24 |
| 80P15-RP2 | F: AACAACAGACTGGTTCCAAA  R: GGTGGTGTCATCTGCATATC | 178 | 55 | 80P15;134E15 |
| 9I24-T7 | F: GGCCATATTGTTGTCACTTC  R: CTTCCTTGGAGGAGTGTGTC | 285 | 55 | 9I24;80P15 |
| 9I24-RP1 | F: CCAGCACCACTTGTTAAAGA  R: CCTATCAAGCTACCAGCCAT | 327 | 55 | 9I24;291I15 |
| 57E15-T7 | F: AGTCAGTGATGCCATCCAGC  R: CTGCAAGGAGATCCAACCAG | 207 | 60 | 57E15;72M13 |
| 57E15-RP2 | F: CTGACTCTTGCTGCTCCTTG  R: CCACTCCAGTGTTCTTGCCT | 232 | 60 | 57E15;190N9 |
| 190N9-T7 | F: TGTAGCTTGCCAGGTTCCTC  R: TATCAGGCTCCTCCGTCCAT | 304 | 60 | 190N9;211N8;  133M1 |
| 190N9-RP2 | F: GTGTCTCTGCAGCATGCCTA  R: GTCACATTGATCGAAGGCAC | 315 | 60 | 190N9;57E15 |
| 211N8-T7 | F: ACCCAGTGCCTTTCTAAACC  R: CCATGTGAATGCAGAGTTCC | 291 | 60 | 211N8;291M9 |
| 211N8-RP2 | F: CCTGGTAGCTCAGCTGGTAA  R: ACTGGAGTGAGCTGACATGC | 224 | 58 | 211N8;133M1;  190N9 |
| 351H10-T7 | F: CAGCTAAACGATTCTGGGTC  R: AGGATGATGGTGAGTGTTGC | 233 | 58 | 351H10;291M9 |
| 351H10-RP2 | F: CTTGGAGCAAGGAGAAGAGC  R: GAACTGAGCGCATGTGATGA | 277 | 60 | 351H10;79L8 |
| 75J19-T7 | F: GGAGTGAAGGTGCTGGTGAG  R: GGCCAGTGGAGGAGACATAA | 317 | 60 | 75J19;290J19 |
| 75J19-RP2 | F: GCCTGGCAGAGCCTTATGTA  R: CACGATGCTGAAGGAAGGAC | 338 | 60 | 75J19;166L22;  114B12 |
| 166L22-T7 | F: GCTAGACAGGGAGATTTGTAA  R: GGAAGAATACAGCAGAGAGC | 243 | 55 | 166L22;167I8 |
| 166L22-RP2 | F: TTGCTGTAGCATTGCCTTTC  R: GTGCCTGGTTATTCTGCTGA | 399 | 60 | 166L22;114B12;  75J19 |
| 167I8-T7 | F: GATCTGGAGATGGTTGCGTA  R: CAGGCAGAATGTGAAGCTGA | 407 | 60 | 167I8;216D18 |
| 167I8-RP1 | F: AATGCAAGCTGTCCAGGTTC  R: AGCCTTCTTGTGGTCTTGGT | 219 | 60 | 167I8;166L22 |
| 216D18-T7 | F: CAGTAATCTGGGCTGTAGGT  R: ACGAGGACAATCTCAGAGAC | 416 | 55 | 216D18;167I8 |
| 216D18-RP2 | F: ATGGCCTGAACTGGTGTTCC  R: CAATGCCATCTCCGATTCATA | 286 | 60 | 216D18;103F21 |
| 103F21-T7 | F: GTCAAGTCATGATGCCGCAC  R: GGTTGCTGAGGCTGGTTCTA | 349 | 60 | 103F21;266O16;  255G2 |
| 103F21-RP2 | F: CAGCAGTAGGATGGAAGGAG  R: CAACATGGACGCAACTAGAG | 562 | 55 | 103F21;216D18 |
| 266O16-T7 | F: CACACATGACTGAACGACTG  R: CAGCTGATCATCTGGTATGG | 341 | 55 | 266O16;288I23 |
| 266O16-RP2 | F: CGCGACACACACACACTTCT  R: CATCAGGTGGCAAGTATTGG | 230 | 60 | 266O16;255G2;  103F21 |
| 288I23-T7 | F: CTGGAAGATTCCTTGTGGCT  R: GTTGTGATGACCTATAAGGGAAA | 290 | 60 | 288I23;53D7 |
| 288I23-RP2 | F: GCCCTCCCTGATTCTAACAA  R: TCCTCCAGAACCATCCATGA | 343 | 60 | 288I23;266O16 |
| 230K5-T7 | F: CAGACAGAGCAAGCTGCACA  R: GAGCACAGGCTTCAGCAGTT | 416 | 60 | 230K5;226G4 |
| 230K5-RP2 | F: GTGCAGCATGCAGGATCTTA  R: AGGAAGGCTCATGGAGGATA | 209 | 60 | 230K5;161A23 |
| 161A23-T7 | F: ACGGAGTATGTGGGTATTTG  R: GCCTAACTACTGCCATGTGT | 206 | 55 | 161A23;230K5 |
| 161A23-RP2 | F: AGATGTGGAGCAGAGTAGGA  R: CCTGGGAATCAGTGTTCTAA | 385 | 55 | 161A23;167J23 |
| 167J23-T7 | F: ACGGAGTATGTGGGTATTTG  R: GCCTAACTACTGCCATGTGT | 206 | 55 | 167J23;277G10 |
| 167J23-RP2 | F: AATGGCGTAAGGAGGGAGTC  R: CCACTGTAGGCGCTCACATT | 598 | 60 | 167J23;161A23 |
| 277G10-T7 | F: TGAGATATGCTGCTGCCTTG  R: GGATTCTTCACCAGCTGACC | 522 | 60 | 277G10;167J23 |
| 277G10-RP1 | F: CCTGGTACTCTGCACACTCC  R: GAATCGCAGTCTCCTGAGGT | 389 | 60 | 277G10;277H11 |
| 277H11-T7 | F: TGAGATATGCTGCTGCCTTG  R: TACGTCTTCTGCATTGCCTG | 552 | 60 | 277H11;277G10 |
| 277H11-RP2 | F: ACGTGTCTGACAGCACCTTG  R: GTTGAGTTGGTGGCACAGAG | 362 | 60 | 277H11;164H22 |
| 74E17-T7 | F: CCTGATCCTTCAGCCTCATT  R: CAGATGTTGTCAGATCCGCA | 263 | 60 | 74E17;168E20;  270P6 |
| 168E20-T7 | F: TCTAGATTCTCACGCCTACC  R: TGGCTCCTCTAGTGAGACAA | 231 | 55 | 168E20;270P6;  74E17 |
| 168E20-RP2 | F: CTGTGTGACCTTGGACAAGT  R: CGATGGATGTGAGTCTGAGT | 523 | 55 | 168E20;102M1 |
| 13P23-T7 | F: AAGGCCAATCAGGTATGCAG  R: GACCACACAGCGAGGATGAG | 344 | 60 | 13P23;102M1 |
| 13P23-RP2 | F: CTTCCGCATCCATACCTGTT  R: TTGCCATGGAGTGATGAGAC | 471 | 60 | 13P23;139B24 |
| 139B24-T7 | F: AGAAGAAGAGGAGGTGGAGA  R: CCTGTGTGAGAGTGGAGTGT | 351 | 55 | 139B24;183A23;  2C18 |
| 139B24-RP1 | F: ATTCGTGGAAGCATCAGAGC  R: GGAGCCTGGTCATCATGAAT | 344 | 60 | 139B24;13P23 |
| 183A23-T7 | F: CTCAGGACAGAACCACTGCT  R: GGCACTGTAACTGGACAAGG | 327 | 58 | 183A23;2C18;  139B23 |
| 183A23-RP1 | F: AGGTCCATCCATGTTGCTTG  R: TAGCACAGACCTGGCCATTC | 410 | 60 | 183A23;2C18;  199K7 |
| 199K7-T7 | F: GCCTCAGAGAGCTTATGTGT  R: CTGCTACGTTCACTTGACCT | 342 | 55 | 199K7; 183A23;2C18 |
| 199K7-RP1 | F: GGATTATCACCAGCCACCAA  R: TGCAATTTACAGCTACGCCA | 309 | 60 | 199K7;230E24;  146K4 |
| 90C20-T7 | F: TGCTCAATCGTGTCCAACTC  R: TGGAAGGACTGATGCTGAAG | 239 | 60 | 90C20;146K4 |
| 90C20-RP2 | F: TGCCTGACTATGTGACCTGG  R: AGTGAAGTCGCTCAGTCGTG | 314 | 60 | 90C20;127J19 |
| 127J19-T7 | F: CATGACAGGAGCAGCTCTCT  R: GAGTTCAGTGCACCTTCACC | 494 | 58 | 127J19;271H22 |
| 127J19-RP2 | F: TGCTGGCTCTGGAACATCTT  R: CCATGGTCTTCCAGGTTCAA | 417 | 60 | 127J19;90C20 |
