# Supplementary material for: A physical map of a BAC clone contig covering the entire autosome insertion between ovine MHC Class IIa and IIb
Source: BMC Genomics. 2012 Aug 16;13:398. doi: 10.1186/1471-2164-13-398 (PMC3475007; doi:10.1186/1471-2164-13-398)
Supplement: Additional file 2 — Figure S1. A complete physical map of entire ovine MHC with the insertion region between class IIa and IIb included. Order and orientation of overlapping BAC clones were jointly determined by combinations of DNA fingerprinting, BAC-end sequencing, and sequence-specific PCR. Genes identified by BAC-end sequencing are marked with erect black lines, with their names listed above. A horizontal bar stands for individual BAC with its identification marked above. Red, purple and green color represent the MHC class I, class III, and class II, representatively. [file 1471-2164-13-398-S2.doc]

Figure S1.


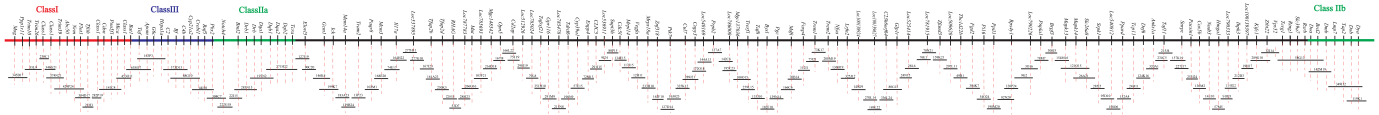


**Figure S1. A complete physical map of entire ovine MHC with the insertion region between class IIa and IIb included.** Order and orientation of overlapping BAC clones were jointly determined by combinations of DNA fingerprinting, BAC-end sequencing, and sequence-specific PCR. Genes identified by BAC-end sequencing are marked with erect black lines, with their names listed above. A horizontal bar stands for individual BAC with its identification marked above. Red, purple and green color represent the MHC class I, class III, and class II, representatively.
